# Tuberculous Abscesses in the Head and Neck Region

**DOI:** 10.3390/diagnostics12030686

**Published:** 2022-03-11

**Authors:** Lukas D. Landegger

**Affiliations:** Department of Otorhinolaryngology, Vienna General Hospital, Medical University of Vienna, 1090 Vienna, Austria; lukas.landegger@meduniwien.ac.at

**Keywords:** tuberculosis, abscess, scrofula, head and neck, epiglottis, larynx, airway, mycobacteria, surgical drainage

## Abstract

Tuberculosis represents a global health challenge and is one of the leading infectious killers, with over a million people succumbing to it every year. While the disease is primarily prevalent in developing countries, where 95% of cases and deaths occur, doctors around the globe need to be able to recognize its diverse clinical manifestations in order to initiate appropriate treatment early. The granulomatous infection caused by *Mycobacterium tuberculosis* typically affects the lungs, but isolated abscesses in the head and neck region can be a less common presentation of the disease, potentially resulting in dysphagia, odynophagia, voice changes, neck swelling, bone erosion, and even life-threatening respiratory distress requiring tracheostomy. Here, characteristic imaging findings and potential surgical options are discussed.

**Figure 1:**
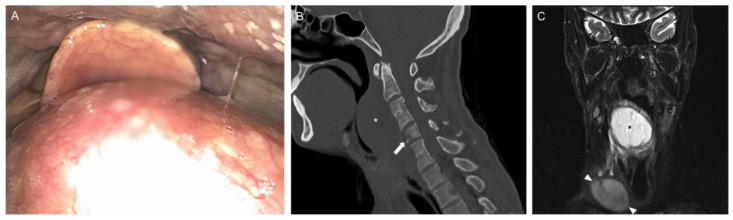
A 30-year-old male Somali refugee presented to the otorhinolaryngology outpatient clinic with an indolent 7 cm × 3 cm right supraclavicular mass. The patient reported a weight loss of 10 kg in 6 months, for which comprehensive diagnostic work-up for infectious/neoplastic etiologies had been initiated two months earlier. This had included an ultrasonographic evaluation and a cervical MRI, which confirmed the size and location of the mass suggestive of a lymph node conglomerate. An unremarkable chest X-ray and serologic screening for HIV, HSV, VZV, CMV, EBV, adenovirus, enterovirus, coxsackievirus, parvovirus, rubella, measles, mumps, hepatitis A, B, and C could not explain the lymphadenopathy. A core needle biopsy of the conglomerate showed granulomatous inflammation with a positive QuantiFERON-TB followed by negative mycobacterial culture/PCR. During a follow-up appointment where these results were discussed, the patient mentioned that he had developed two-week-long progressive hoarseness and subtle dyspnea. Flexible endoscopy revealed midline pharyngeal bulging pushing the epiglottis anteriorly (**Panel A** and Video S1). Cervical spine CT (**Panel B**, sagittal) showed the new retropharyngeal mass (5 cm × 3.5 cm, asterisk) and erosion of cervical vertebrae (mainly C5, arrow). As opposed to the imaging carried out two months earlier, the repeat cervical MRI (**Panel C**, coronal, STIR sequence) visualized both retropharyngeal abscess (asterisk) and supraclavicular conglomerate (arrowheads). Not all sequences could be obtained as the patient developed dyspnea during the MRI examination. Awake tracheostomy under local anesthesia, excision of the conglomerate, and repeated transoral/transcutaneous abscess drainage ensued. Positive culture/PCR confirmed pansensitive *Mycobacterium tuberculosis* and quadruple anti-TB treatment was initiated. The patient tolerated the therapy without adverse events and is doing well 6 months later with final orthopedic repair remaining. In retrospect, some of the vertebral lesions could have been observed in the initial cervical MRI carried out at an external diagnostic imaging center, but the local radiologist in private practice did not explicitly comment on them as the focus was the lymph node conglomerate. The primary infection for the hematogenous spread remains unknown. This case highlights that the differential diagnosis of TB has to be considered in all atypical head and neck pathology. Scrofula, i.e., tuberculous lymphadenitis in the cervical region, is one of the most common extrapulmonary manifestations of TB [1]. Response to antimycobacterial therapy is known to be slower than with pulmonary lesions, with novel treatment regimens yet to demonstrate efficacy [2]. In the case of abscess formation, different surgical options have been analyzed over many decades and have been found to be efficacious in combination with drug treatment [3]. Tuberculous spondylitis, or Pott disease, can lead to degeneration and even caseous necrosis of intervertebral discs, as can be seen between C3/4 and C6/7 above, or lytic destruction of vertebrae, as highlighted in C5 [4]. Globalization can result in the emergence and reemergence of infectious diseases such as TB in low-incidence countries [5]. Thus, knowledge of the signs and symptoms to establish a diagnosis is crucial to guarantee that patients receive adequate and timely treatment.

## Data Availability

Not applicable.

## Supplementary Materials

The following supporting information can be downloaded at: https://www.mdpi.com/article/10.3390/diagnostics12030686/s1, Video S1: Transnasal endoscopy.
